# 

**Published:** 1984-07

**Authors:** 


Contents

77. **The Search for an Ideal Wound Dressing**
Sean S. Brennan and David J. Leaper

80. **Feasibility of Direct Access Flexible Fibreoptic Sigmoidoscopy for General Practice**
K. D. Vellacott

82. **The Clinical Investigation Unit, Ham Green Hospital**
Angela M. Shepherd

84. **Fulminating Aspergillus Pneumonia Complicating Radiation Fibrosis**
D. W. Pitcher, P. Wood, P. R. Goddard and J. R. Rees

88. **'Lot's Wife' Syndrome in Acute Myeloid Leukaemia**
M. M. Barnett

90. **Pulsed Doppler Echocardiography in the Diagnosis of Ventricular Septal Defect and Patent Ductus Arteriosus**
Graham Buirski and Peter Wilde

95. **From our Correspondents**

98. **From our Foreign Correspondent**

100. **Book Reviews**

102. **Obituary**

